# Contribution of blood-brain barrier-related blood-borne factors for Alzheimer’s disease vs. vascular dementia diagnosis: A pilot study

**DOI:** 10.3389/fnins.2022.949129

**Published:** 2022-08-08

**Authors:** Min Gong, Jianping Jia

**Affiliations:** ^1^Innovation Center for Neurological Disorders and Department of Neurology, Xuanwu Hospital, Capital Medical University, National Clinical Research Center for Geriatric Diseases, Beijing, China; ^2^Beijing Key Laboratory of Geriatric Cognitive Disorders, Beijing, China; ^3^Clinical Center for Neurodegenerative Disease and Memory Impairment, Capital Medical University, Beijing, China; ^4^Center of Alzheimer’s Disease, Beijing Institute of Brain Disorders, Collaborative Innovation Center for Brain Disorders, Capital Medical University, Beijing, China; ^5^Key Laboratory of Neurodegenerative Diseases, Ministry of Education, Beijing, China

**Keywords:** vascular dementia (VaD), Alzheimer’s disease (AD), blood-brain barrier, low-density lipoprotein receptor-related protein, cyclophilin A, matrix metalloproteinase-9

## Abstract

**Background:**

Alzheimer’s disease (AD) and vascular dementia (VaD) are the two most common types of neurodegenerative dementia among the elderly with similar symptoms of cognitive decline and overlapping neuropsychological profiles. Biological markers to distinguish patients with VaD from AD would be very useful. We aimed to investigate the expression of blood-brain barrier (BBB)-related blood-borne factors of soluble low-density lipoprotein receptor-related protein 1 (sLRP1), cyclophilin A (CyPA), and matrix metalloproteinase 9 (MMP9) and its correlation with cognitive function between patients with AD and VaD.

**Materials and methods:**

Plasma levels of sLRP1, CyPA, and MMP9 were analyzed in 26 patients with AD, 27 patients with VaD, and 27 normal controls (NCs). Spearman’s rank correlation analysis was used to explore the relationships among biomarker levels, cognitive function, and imaging references. Receiver operating characteristic (ROC) curve analysis was used to discriminate the diagnosis of AD and VaD.

**Results:**

Among these BBB-related factors, plasma CyPA levels in the VaD group were significantly higher than that in the AD group (*p* < 0.05). Plasma sLRP1 levels presented an increasing trend in VaD while maintaining slightly low levels in patients with AD (*p* > 0.05). Plasma MMP9 in different diagnostic groups displayed the following trend: VaD group > AD group > NC group, but the difference was not statistically significant (*p* > 0.05). Furthermore, plasma sLRP1 levels were positively related to MoCA scores, and plasma CyPA levels were significantly correlated with MTA scores (*p* < 0.05) in the AD group. Plasma MMP9 levels were negatively correlated with MoCA scores (*p* < 0.05) in the VaD groups. No significant correlation was detected between the other factors and different cognitive scores (*p* > 0.05). ROC analysis showed a good preference of plasma CyPA [AUC = 0.725, 95% CI (0.586–0.865); *p* = 0.0064] in diagnosis.

**Conclusion:**

The plasma CyPA level is a reference index when distinguishing between an AD and subcortical ischemic vascular dementia (SIVD) diagnosis. Blood-derived factors associated with the BBB may provide new insights into the differential diagnosis of neurodegenerative dementia and warrant further investigation.

## Introduction

Dementia manifests as global cognitive decline and significantly impairs daily activities, which has imposed a heavy burden on the public and healthcare systems as society ages (Jia et al., 2020). Alzheimer’s disease (AD) and vascular dementia (VaD) are the two most common types of neurodegenerative dementia among the elderly. AD has a slow course characterized by gradual deterioration of cognitive function. VaD has a variable course presented by a stepwise worsening of executive function, which can have a sudden or slow onset (O’Brien and Thomas, 2015). Although these two types of dementia share many clinical features, including symptoms of cognitive decline and overlapping neuropsychological profiles, the underlying pathophysiological mechanisms are different. Efficient therapy depends on accurate diagnosis, thus it is crucial to distinguish between VaD and AD (Neto et al., 2015; Tachibana et al., 2016). Despite advances in molecular neuroimaging, the understanding of clinicopathological relevance and the development of novel biomarkers have been limited in the last decade (Park and Moon, 2016; Bjerke and Engelborghs, 2018). Moreover, imaging is relatively expensive and invasive and is usually not immediately available for such a diagnosis. As another option, lumbar puncture is an invasive procedure, which requires written informed consent. Clinicians still need reliable and non-invasive molecular markers for the differential diagnosis of neurodegenerative dementia. Blood testing is an economical, minimally invasive and more accessible procedure and is more suitable for investigating these pathological mechanisms and distinguishing between different forms, at least as a primary screening test.

Although the etiology of AD and VaD may differ, the overall mechanisms of subsequent neurovascular dysfunction are similar, with defective blood-brain barrier (BBB) function. BBB failure is considered to be a core mechanism in vascular-related diseases and neurodegenerative dementia, driving disease pathology and progression (Yamazaki and Kanekiyo, 2017; Cai et al., 2018; Ueno et al., 2019; Uemura et al., 2020). The breakdown of the BBB is caused by the degeneration of pericytes and endothelial cells, loss of tight junctions, and brain capillary leakages, which cause toxic molecules from the blood to enter the brain and initiate multiple neurodegenerative pathways (Bell et al., 2010; Zlokovic, 2011; Nelson et al., 2016; Storck et al., 2016; Montagne et al., 2020). Currently, it is speculated that low-density lipoprotein receptor-related protein 1 (LRP1), cyclophilin A (CyPA), and matrix metallopeptidase 9 (MMP9) are involved in the regulation of BBB permeability (Bell et al., 2012; Halliday et al., 2016). Soluble LRP1 (sLRP1) circulates freely in plasma and is primarily responsible for peripheral Aβ clearance (Quinn et al., 1997). Several studies have reported a significant reduction in LRP1 expression in brain microvascular endothelial cells in AD (Shibata et al., 2000; Deane et al., 2004; Donahue et al., 2006). Circulating sLRP in the plasma binds Aβ and prevents brain reentry across the BBB, producing a peripheral sink that promotes the outflow of Aβ from the brain (Sagare et al., 2007, 2012; Deane et al., 2008). Plasma sLRP1 levels and Aβ binding to sLRP1 are significantly reduced due to increased levels of oxidized sLRP1, which does not bind Aβ, resulting in an increase in free Aβ levels in plasma to return to the brain *via* the receptor for advanced glycation end products (RAGE) in patients with AD (Deane et al., 2003; Sagare et al., 2007, 2011). CyPA is secreted by activated macrophages, lymphocytes, and platelets and mediates the harmful effects of pericytes on BBB disruption (Seizer et al., 2010, 2015, 2016; Pan et al., 2020). Previous studies have shown that the plasma CyPA level is a new biomarker for the diagnosis of coronary artery disease (CAD) and renal disease progression and is used as a prognostic factor in patients with ruptured intracranial aneurysms (Satoh et al., 2013; Ramachandran et al., 2014; Kao et al., 2015; El-Ebidi et al., 2020; Rath et al., 2020). MMP9 is involved in the increase of BBB permeability during AD, which accelerates its onset (Barr et al., 2010; Shackleton et al., 2019). These results provide support for the use of BBB-related blood-borne factors as sensitive predictors of neurodegenerative dementia (specifically BBB dysfunction-related cognitive decline) because they are tentatively present in biofluids and are involved in BBB function. Few studies have analyzed the correlation between BBB-related blood-borne factors (sLRP1, CyPA, and MMP9) and dementias.

In this clinical study, we aimed to measure plasma sLRP1, CyPA, and MMP9 levels in patients with AD, patients with VaD, and healthy controls and to evaluate their correlation with cognitive function to assist the discovery of new biomarkers.

## Materials and methods

### Study populations

A total of 80 participants were enrolled in our research who were admitted to the Department of Xuanwu Hospital, Capital Medical University from July 2019 to July 2021. More specifically, 26 patients with AD, 27 patients with subcortical ischemic vascular dementia (SIVD), and 27 age-matched cognitive normal controls (NC) were recruited. The diagnosis of AD was performed according to the National Institute on Aging and the Alzheimer’s Association (NIA-AA) criteria (Jack et al., 2011). The diagnosis of SIVD was performed according to the modified National Institute of Neurological Disorders and Stroke and the Association Internationale pour la Recherche et l’Enseignement en Neurosciences (NINDS-AIREN) and has evidence of ischemic lesions on brain magnetic resonance imaging (Erkinjuntti, 2003). Moreover, healthy individuals, not affected by neurodegenerative diseases, were recruited as NC. This study was approved by the ethics committee. Written informed consent was obtained before enrollment. The details of inclusion/exclusion criteria for AD and VD are shown in the Supplementary material.

### Plasma sample processing

Blood samples were taken in the morning after a 12-h fast. Notably, 6 ml of whole blood were drawn from each subject and stored in a polypropylene tube containing EDTA. Plasma separation was performed by centrifugation at 1,880 × *g* for 15 min. Finally, plasma was collected in centrifugal tubes and stored at −80°C until analysis.

### Measurements

The levels of sLRP1, CyPA, and MMP9 were measured in plasma using a commercially available enzyme-linked immunosorbent assay (ELISA) (LRP1: IC-LRP1-Hu, ImmunoClone, United States; CyPA: KE1726, immunoway, United States; and MMP9: KE1407, immunoway, United States) according to the manufacturer’s instructions, and the dilution concentrations of sLPR1, CYPA, and MMP9 were 1:2,000, 1:10, and 1:50, respectively. The cognition of participants was assessed using the Mini-Mental State Examination (MMSE) and the Montreal Cognitive Assessment (MoCA). Total cholesterol (TC), triglyceride (TG), low-density lipoprotein cholesterol (LDL), and high-density lipoprotein cholesterol (HDL) were measured by the clinical levorotary testing center. Periventricular hyperintensity (PVH) and deep white matter hyperintense signals (DWMH) were evaluated using the Fazekas scale, and the scores of these two parts were summarized to obtain the total score (the lowest total score is 0, and the highest is 6) (Fazekas et al., 1987). A high Fazekas score is clinically associated with the diagnosis of individuals at high risk of cerebrovascular disease (Hilal et al., 2021). Concordantly, a high Fazekas score can successfully predict the cognitive function of patients with dementia in a clinical setting (Lam et al., 2021). Another, medial temporal lobe atrophy (MTA) visual rating scale has been shown to have high diagnostic accuracy for AD (Cavedo et al., 2014). It has also been reported in patients with VaD (Barber et al., 2000). In our study, ratings of PVH (0 = absence, 1 = “caps” or pencil-thin lining, 2 = smooth “halo,” and 3 = irregular) and DWMH (0 = absence, 1 = punctate foci, 2 = beginning confluence of foci, and 3 = large confluent areas) were summarized into total Fazekas scores using the axial fluid-attenuated inversion recovery (FLAIR) images from a 3T MRI and were classified into low WMSA burden (Fazekas scores < 3) and high WMSA burden (Fazekas scores ≥ 3). MTA was dichotomized into groups of 0–1 (none to mild) vs. 2–4 (moderate to severe) by analyzing the width of the choroidal fissure, the width of the temporal horn of the lateral ventricle, and the height of the hippocampus on T1-weighted coronal sections. The total score of each patient was approved by two experienced neurologists who were blind to the clinical data. The typical images of MTA/white matter hyperintensity from patients have shown in Supplementary Figure 1.

### Statistical analysis

Descriptive statistics were used to summarize the participant characteristics. For normally distributed data, including the variables of age, TC, HDL, MMSE scores, and MoCA scores, the means ± standard deviation (SD) was used to describe the quantitative variables. The two groups were compared using *t*-test, among groups were compared using ANOVA, and the differences between the groups were statistically significant based on further pairwise comparison *t*-test with normal distributions. For non-normally distributed data, including the variables of TG, LDL, years of education, sLRP1, CyPA, and MMP9 levels, we used medians and interquartile ranges to describe the quantitative variables. Baseline characteristics were compared using the independent-sample Kruskal-Wallis test. Categorical data were presented as proportions, and among groups were compared using the chi-square test. The Spearman correlation analysis was performed to assess the correlation between two quantitative variables, adjusted for age, sex, and education. The diagnostic value of AD and SIVD was estimated by the area under the curve (AUC) using the receiver operating characteristic (ROC) curve. *p* < 0.05 was considered to indicate significant results. *p*-values were corrected using the Bonferroni method for multiple comparison corrections. All statistical analyses were performed using SPSS version 23.0 (SPSS Inc., Chicago, IL, United States).

## Results

### Demographics

The demographic and clinical characteristics of each diagnostic group are described in Table 1. No significant difference was found between the groups in age, gender distribution, education, and others. The differences in the MMSE, MoCA scores, and Fazekas WMSA burden among the different groups were presented (refer to Table 1).

**TABLE 1:** Participant characteristics.

Characteristics	AD (*n* = 26)	SIVD (*n* = 27)	NC (*n* = 27)	*P*
Age, Mean ± SD (year)	66.65 ± 9.53	65.37 ± 9.93	65.04 ± 6.12	0.708
Male, *N* (%)	11(42.3)	18 (66.7)	10 (37.0)	0.068
Education, median (Q25, Q75) (year)	12.00 (9.00, 15.00)	12.00 (9.00, 13.00)	12.00 (9.00, 12.00)	0.163
TC, Mean ± SD (mmol/L)	4.50 ± 1.09	4.07 ± 1.46	4.06 ± 0.87	0.262
TG, median (Q25, Q75) (mmol/L)	1.20 (1.01, 1.74)	1.34 (1.03, 1.72)	1.02 (0.79, 1.38)	0.060
LDL, median (Q25, Q75) (mmol/L)	2.55 (1.92, 3.22)	1.77 (1.35, 3.18)	2.52 (1.83, 2.95)	0.414
HDL, Mean ± SD (mmol/L)	1.32 ± 0.38	1.20 ± 0.43	1.25 ± 0.38	0.537
Hypertension, *N* (%)	11 (42.3)	17 (63.0)	20 (74.1)	0.057
Diabetes, *N* (%)	6 (23.1)	7 (25.9)	9 (33.3)	0.687
Hyperlipidemia, *N* (%)	3 (11.5)	5 (18.5)	9 (33.3)	0.139
MMSE, Mean ± SD (score)	19.12 ± 5.69**^#^**	21.09 ± 8.32**^#^**	27.63 ± 1.28	<0.001
MoCA, Mean ± SD (score)	13.60 ± 4.87**^#^**	16.27 ± 7.32**^#^**	25.07 ± 1.04	<0.001
Fazekas WMSA burden, *N* (%)				<0.001
High (3–6 scores)	1 (3.8)*	23 (85.7)	NA	
Low (0–2 scores)	25 (96.2)	4 (14.3)	NA	
MTA, *N* (%)				0.128
None to mild (0–1 scores)	8 (30.8)	14 (52.2)	NA	
Moderate to severe (2–4 scores)	18 (69.2)	13 (47.8)	NA	

Data are shown as mean ± SD and N (%).

*Compared with the subcortical ischemic vascular dementia (SIVD) group, the difference was significant (*p* < 0.05).

^#^Compared with the NC group, the difference was significant (*p* < 0.05).

### Differences in sLRP1, CyPA, and MMP9 levels between diagnostic groups

**FIGURE 1:**
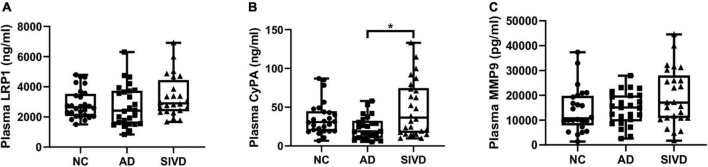
Box and dot plots of plasma **(A) s**LRP1, **(B)** CyPA, and **(C)** MMP9 per diagnostic group. Analytes with significant differences between diagnostic groups are shown. *p = 0.018.

Plasma sLRP1, CyPA, and MMP9 levels in the diagnostic group are shown in Table 2. Plasma sLRP1 levels in the AD group showed a decreasing trend compared with that in the NC group, but the difference was not statistically significant (*H* = 3.817, *p* = 0.148). The difference in plasma CyPA levels was statistically significant across the three groups (*H* = 8.302, *p* = 0.016). Further pairwise comparison showed that plasma CyPA levels in the SIVD group were significantly higher than that in the AD group (*p* = 0.018). The differences in the plasma MMP9 levels were not statistically significant (*H* = 3.778, *p* = 0.151). Figure 1 shows the plasma sLRP1, CyPA, and MMP9 levels in each group.

**TABLE 2:** sLRP1, CyPA, and MMP9 values by diagnostic groups.

Characteristics	AD (*n* = 26)	SIVD (*n* = 27)	NC (*n* = 27)	H	*P*
sLRP1, median (Q25, Q75) (ng/ml)	2409.65 (1544.57, 3732.41)	2896.88^a^ (2353.69, 4694.35)	2639.14 (2042.29, 3564.04)	3.817	0.148
CyPA, median (Q25, Q75) (ng/ml)	18.95*^b^ (10.46, 35.05)	36.57 (17.51, 73.32)	30.78^b^ (21.00, 49.15)	8.302	0.016
MMP9, median (Q25, Q75) (pg/ml)	15076.43^c^ (9873.41,20403.41)	17076.83 (10679.13, 26947.31)	10699.70*^c^ (8450.16, 20368.37)	3.778	0.151

Data are shown as medians (IQR).

*Compared with the subcortical ischemic vascular dementia (SIVD) group, the difference was significant (*p* < 0.05); ^a^Delete 2 Outliers; ^b^Delete 3 Outliers; ^c^Delete 5 Outliers. *p < 0.05.

### The correlation of plasma biomarker levels with cognition score

Spearman correlation analysis found that plasma sLRP1 levels were positively related to MoCA (*r* = 0.414, *p* = 0.040) scores in the AD groups, and plasma MMP9 levels and MoCA (*r* = −0.528, *p* = 0.014) scores showed a significant negative correlation in the SIVD groups; however, CyPA levels were not significantly correlated with cognitive scores (Table 3 and Supplementary Figure 2). Correlation analysis showed a significant correlation between plasma CyPA levels and MTA scores in the AD group (*r* = 0.464, *p* = 0.017). No significant correlation was detected between the other biomarkers and imaging parameters (*p* > 0.05) (Table 3). The correlation of plasma biomarkers with other factors is shown in the Supplementary Tables 1–4.

**TABLE 3:** Correlation of plasma biomarker levels with cognitive scores and imaging parameters.

Factors	sLRP1		CyPA		MMP9	
			
	r	*P*	r	*P*	r	*P*
**The whole sample**						
MMSE	0.167	0.152	0.066	0.571	−0.128	0.276
MoCA	0.169	0.149	0.077	0.514	−0.182	0.123
Fazekas	0.089	0.543	0.185	0.203	0.151	0.309
MTA	−0.010	0.945	−0.086	0.558	−0.007	0.961
**The AD groups**						
MMSE	0.303	0.141	0.085	0.685	0.108	0.606
MoCA	0.414	0.040*	0.237	0.254	−0.017	0.937
Fazekas	0.053	0.797	0.259	0.201	0.167	0.416
MTA	0.346	0.083	0.464	0.017*	0.000	1.000
**The SIVD groups**						
MMSE	0.314	0.145	−0.139	0.527	−0.325	0.140
MoCA	0.228	0.308	−0.397	0.067	−0.528	0.014*
Fazekas	0.001	0.998	0.195	0.398	−0.248	0.278
MTA	0.197	0.367	−0.157	0.476	0.142	0.529

Adjusted for age, sex, and education.

*The correlation of plasma biomarker levels with cognitive scores and imaging parameters, the difference was significant (*p* < 0.05).

### Differential diagnostic value of CyPA concentrations

**FIGURE 2:**
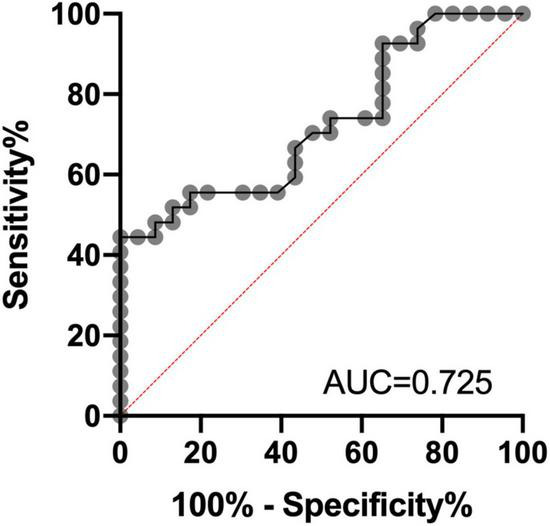
Receiver operating characteristic (ROC) analysis of CyPA concentrations in plasma as a candidate biomarker of differential diagnosis for Alzheimer’s disease (AD) and subcortical ischemic vascular dementia (SIVD). AUC, area under the curve.

The ROC analysis was finally performed to assess the differential diagnosis value in patients with AD and SIVD group using CyPA concentration; the performance was found to be good [AUC = 0.725, 95% CI (0.586–0.865); *p* = 0.0064]. ROC analysis identified a cutoff value for CyPA of 60.15 ng/ml (Figure 2).

## Discussion

Our study aimed to explore the levels of BBB-related blood-borne factors sLRP1, CyPA, and MMP9 in AD, SIVD, and NC. Furthermore, the association of BBB-related factors with cognitive function and imaging parameters was evaluated. To the best of our knowledge, this study is the first to demonstrate that BBB-related blood-borne factors are associated with different types of dementia. Our results support the use of blood-borne factors as potential biomarkers for the differential diagnosis of dementia.

This study showed that plasma CyPA levels presented a decreasing trend in the AD group, and an increasing trend in the SIVD group compared with the NC group, and the plasma CyPA level in the SIVD group was significantly higher than that in the AD group. We used plasma CyPA as a reference index for the differential diagnosis of SIVD and AD. To date, studies have shown that CyPA secretion is increased in patients with central nervous system diseases, such as cerebrovascular disease, brain trauma, and obstructive sleep apnoea with mild cognitive impairment (MCI) (Redell et al., 2007; Chang et al., 2018, 2020; Li et al., 2021). These studies have confirmed that CyPA is critical for brain damage. Furthermore, CyPA levels in endothelial cells and pericytes of the brain are elevated in patients with AD (Halliday et al., 2016). Associations between serum CyPA levels and regional gray matter volume indicated that blood levels of CyPA may reflect the pathological mechanism of AD in the brain (Choi et al., 2021). Given our study of CyPA, we postulate that plasma CyPA exerts a crucial association with dementia, especially when comparing AD vs. VaD. The molecular mechanisms underlying the effects of CyPA on AD and SIVD are not yet fully understood. *In vitro* studies have shown that CyPA induces endothelial dysfunction, the proliferation of vascular smooth muscle cells, and the migration of inflammatory cells and promotes the development of atherosclerosis (Nigro et al., 2011; Satoh et al., 2011). Pan et al. (2020) demonstrated that CyPA mediates the destruction of brain BBB by pericytes through CD147/NF-κB/MMP9 signaling and junction protein degradation, which may provide a new insights into the management by targeting CyPA and pericytes. *APOE* protein can control BBB integrity *via* the inhibition of CypA-MMP9 signaling cascades (Halliday et al., 2016; Montagne et al., 2020, 2021). Bell et al. also found that the level of CyPA in the cerebral microvessels of *APOE* ε*4* and *APOE*−/− mice was five to six times higher than that in the control group of *APOE* ε*2* and *APOE* ε*3* mice. Knocking out CyPA could eliminate BBB damage caused by *APOE* ε*4* and *APOE*−/− mice. Moreover, treating *APOE* ε*4* mice with low doses of cyclosporine was shown to eliminate BBB damage, indicating that the change in CyPA on the BBB is reversible, and CyPA may be a therapeutic target that causes BBB destruction (Bell et al., 2012; Montagne et al., 2021). Taken together, these results indicate that CyPA controls cerebrovascular integrity. In conjunction with our study, although the number of patients in this study was small, the differences between patients with VaD and AD were significant. These results suggest that plasma CyPA measurements, while not diagnostic, might be combined with psychometric and imaging references to improve the early differentiation between VaD and AD, which may help to better select patients in future clinical trials. To the best of our knowledge, this is the first study to describe elevated plasma CyPA levels in patients with VaD.

Our results provide evidence of differences in plasma CyPA in AD vs. VaD. We also found that plasma CyPA levels were associated with MTA scores in the AD group; however, CyPA levels were not significantly correlated with cognitive scores. The breakdown of the BBB initially occurs in the hippocampus during normal aging, which is a key area for memory. Disruption of the BBB in the hippocampus is associated with MCI, which is in turn associated with damage to pericytes (Montagne et al., 2015). Individuals with early cognitive impairment, regardless of changes in Aβ and/or tau biomarkers of AD, develop brain capillary damage and disruption of the hippocampal BBB, indicating that BBB disruption is an early biomarker of human cognitive dysfunction independent of Aβ and tau (Nation et al., 2019). These results indicate that CyPA is involved in the brain structure of the hippocampus *via* the BBB, which may be an early event in the aging human brain that begins in the hippocampus and is reflected by CyPA. Thus, CyPA may predict early BBB dysfunction in the hippocampus in AD; however, the mechanism requires further exploration. Another study showed that CyPA and MMP9 levels in serum were associated with cognitive impairment and white matter signal abnormalities, which is controversial in our study (Li et al., 2021). The correlation between CyPA levels and cognitive function requires further verification.

Lipoprotein receptor-related protein 1, a major transporter in the brain-to-blood clearance of Aβ across the BBB, is associated with cognitive decline in AD (Deane et al., 2004; Storck et al., 2016; Ma et al., 2018). Our study found that lower plasma sLRP1 levels were associated with cognitive decline in patients with AD. A previous study showed that cannabinoid treatment enhanced Aβ transfer at the BBB, accompanied by increased brain and plasma sLRP1 levels (Bachmeier et al., 2013). This finding is consistent with those of this study. Another study found that BBB-associated pericytes cleared Aβ aggregation through LRP1/*APOE* subtype-specific mechanisms, supporting the role of the LRP1/*APOE* interaction as a potential therapeutic target for controlling Aβ clearance in AD (Ma et al., 2018). Masaya et al. identified LRP1 as the potential molecular mechanism by which *APOE* ε*4* in patients with AD intensifies the deposition of Aβ protein in the brain, which was seen both in a mouse model of AD and in autopsies of patients with AD (Tachibana et al., 2019). Halliday et al. also suggested that *APOE* ε*4* leads to the accelerated loss of the LRP1-dependent CyPA-MMP9 BBB degradation pathway in pericytes and endothelial cells (Halliday et al., 2016; Nikolakopoulou et al., 2021). A recent study suggested that the loss of brain endothelial LRP1 results in the loss of BBB integrity, neuronal loss, and cognitive deficits in mice, which could be reversed by endothelial-specific LRP1 gene therapy (Nikolakopoulou et al., 2021). Thus, increased cerebral LRP1 and plasma sLRP may explain the increased Aβ BBB transport and may provide an effective strategy to reduce the Aβ burden in the AD brain. At present, many studies have focused on the inhibition of Aβ production. However, the occurrence and development of AD are believed to be due to the reduction in Aβ, rather than the excessive production of Aβ (Mawuenyega et al., 2010; Xiang et al., 2015; Wang et al., 2017). Our study focused on Aβ clearance-related proteins and enzymes in AD, which can help discover their potential in the development of AD drugs and provide an optimistic prospect for future therapeutic targets. Furthermore, previous studies found downregulation of LRP1 protein and mRNA expression in VaD rats *via* the IKK/NF-κB signaling pathway (Cai et al., 2020; Wang et al., 2020). Nevertheless, Mercedes et al. proposed that serum sLRP1 can serve as a candidate marker to differentiate AD from mixed dementia phenotypes, with a significant increase in sLRP1 serum protein levels in subjects with mixed dementia and relatively normal levels in AD (Lachén-Montes et al., 2021). Our study also showed that plasma sLRP1 levels presented an increasing trend in VaD while maintaining slightly low levels in patients with AD. This inconsistency may be attributed to the relatively small sample size, different samples, and methods used. Further research is needed to determine whether and how sLRP1 in the blood affects cognitive preference in patients with dementia.

Matrix metallopeptidase 9 is a major protein related to brain disorders and the BBB (Rempe et al., 2016). Inhibition of MMP prevented tight junction protein loss, suggesting that MMP interferes with barrier integrity by degrading tight junction proteins (Yang et al., 2007). Previous studies have found that plasma MMP-9 levels of patients with AD are higher than those in normal controls through an increase in BBB permeability (Lorenzl et al., 2003, 2008). However, Horstmann et al. (2010) showed that plasma MMP9 activity was decreased by 41% in patients with AD compared with that in normal controls. Another study verified that the damage of MMP9 to neurovascular units is thought to be related to vascular cognitive dysfunction by increasing the BBB opening (Candelario-Jalil et al., 2011). The level of MMP9 in the CSF of patients with VaD was higher than that in the AD and normal control groups (Adair et al., 2004). In this study, plasma MMP9 in different diagnostic groups displayed the following trend: SIVD group > AD group > NC group, and higher plasma MMP9 was correlated with cognitive decline, which was broadly consistent with previous studies. MMP9 may increase the permeability of the BBB, activate inflammatory factors, cause various inflammatory responses in cells, and accelerate the onset of disease. Thus, MMP9 may contribute to the course of senile neurodegenerative dementia and may play a role in predicting cognitive function in the periphery. Further studies are required to explore the role of MMP in AD progression.

Our study has some limitations. First, due to the relatively small number of experimental studies and limited research participants, the quality of the statistical analysis may be affected; second, cross-sectional studies have limited causality, and a longitudinal follow-up study is needed to elucidate the relationship between CyPA, sLRP1, and MMP9 levels and disease progression; third, the diagnostic value of a single biomarker is limited, and combinations with other biomarkers should be considered in the future to be used to predict the diagnosis of neurodegenerative dementia; fourth, considering the effect of *APOE* on neurovascular injury and neuronal dysfunction, clarifying the relationship of BBB-related blood-borne factors with *APOE* genotypes is required in the future; fifth, as the assessment of imaging parameters uses visual rating, adding some quantitative volumetric analysis such as hippocampal volume would be informative in future validation studies; the assessment of scales for other cognitive domains and their correlations with biomarkers should also be considered; and finally, we did not have additional information to clarify the mechanism of BBB dysfunction.

In summary, our study suggests that the plasma CyPA level is a reference index when distinguishing between an AD and SIVD diagnosis. sLRP1 and MMP-9 may be ideal biomarkers of cognitive decline. Our findings highlight that blood-derived factors associated with the BBB may provide new insights into the differential diagnosis of neurodegenerative dementia and warrant further investigation.

## Data availability statement

The raw data supporting the conclusions of this article will be made available by the authors, without undue reservation.

## Ethics statement

The studies involving human participants were reviewed and approved by the Ethics Review Board of the Xuanwu Hospital. The patients/participants provided their written informed consent to participate in this study.

## Author contributions

JJ supervised and obtained funding for this study. MG did the statistical analysis. Both authors designed the study, critically revised and drafted the manuscript, contributed to the collection, analysis, and interpretation of data, and approved the final version of the manuscript.

## References

[B1] Adair J. C. Charlie J. Dencoff J. E. Kaye J. A. Quinn J. F. Camicioli R. M. (2004). Measurement of gelatinase B (MMP-9) in the cerebrospinal fluid of patients with vascular dementia and Alzheimer disease. *Stroke* 35 e159–62. 10.1161/01.Str.0000127420.10990.76 15105518

[B2] Bachmeier C. Beaulieu-Abdelahad D. Mullan M. Paris D. (2013). Role of the cannabinoid system in the transit of beta-amyloid across the blood-brain barrier. *Mol. Cell. Neurosci.* 56 255–262. 10.1016/j.mcn.2013.06.004 23831388

[B3] Barber R. Ballard C. McKeith I. G. Gholkar A. O’Brien J. T. (2000). MRI volumetric study of dementia with Lewy bodies: A comparison with AD and vascular dementia. *Neurology* 54 1304–1309. 10.1212/wnl.54.6.1304 10746602

[B4] Barr T. L. Latour L. L. Lee K. Y. Schaewe T. J. Luby M. Chang G. S. (2010). Blood-brain barrier disruption in humans is independently associated with increased matrix metalloproteinase-9. *Stroke* 41 e123–8. 10.1161/strokeaha.109.570515 20035078 PMC2827673

[B5] Bell R. D. Winkler E. A. Sagare A. P. Singh I. LaRue B. Deane R. (2010). Pericytes control key neurovascular functions and neuronal phenotype in the adult brain and during brain aging. *Neuron* 68 409–427. 10.1016/j.neuron.2010.09.043 21040844 PMC3056408

[B6] Bell R. D. Winkler E. A. Singh I. Sagare A. P. Deane R. Wu Z. (2012). Apolipoprotein E controls cerebrovascular integrity via cyclophilin A. *Nature* 485 512–516. 10.1038/nature11087 22622580 PMC4047116

[B7] Bjerke M. Engelborghs S. (2018). Cerebrospinal Fluid Biomarkers for Early and Differential Alzheimer’s Disease Diagnosis. *J. Alzheimers Dis.* 62 1199–1209. 10.3233/jad-170680 29562530 PMC5870045

[B8] Cai H. Cai T. Zheng H. Liu L. Zhou L. Pang X. (2020). The Neuroprotective Effects of Danggui-Shaoyao San on Vascular Cognitive Impairment: Involvement of the Role of the Low-Density Lipoprotein Receptor-Related Protein. *Rejuvenation Res.* 23 420–433. 10.1089/rej.2019.2182 32242481

[B9] Cai Z. Qiao P. F. Wan C. Q. Cai M. Zhou N. K. Li Q. (2018). Role of Blood-Brain Barrier in Alzheimer’s Disease. *J. Alzheimers Dis.* 63 1223–1234. 10.3233/jad-180098 29782323

[B10] Candelario-Jalil E. Thompson J. Taheri S. Grossetete M. Adair J. C. Edmonds E. (2011). Matrix metalloproteinases are associated with increased blood-brain barrier opening in vascular cognitive impairment. *Stroke* 42 1345–1350. 10.1161/strokeaha.110.600825 21454822 PMC3119779

[B11] Cavedo E. Pievani M. Boccardi M. Galluzzi S. Bocchetta M. Bonetti M. (2014). Medial temporal atrophy in early and late-onset Alzheimer’s disease. *Neurobiol. Aging* 35 2004–2012. 10.1016/j.neurobiolaging.2014.03.009 24721821 PMC4053814

[B12] Chang C. S. Kuo C. L. Huang C. S. Cheng Y. S. Lin S. S. Liu C. S. (2020). Association of cyclophilin A level and pulse pressure in predicting recurrence of cerebral infarction. *Kaohsiung J. Med. Sci.* 36 122–128. 10.1002/kjm2.12143 31670477 PMC11896586

[B13] Chang C. S. Su S. L. Kuo C. L. Huang C. S. Tseng W. M. Lin S. S. (2018). Cyclophilin A: A Predictive Biomarker of Carotid Stenosis in Cerebral Ischemic Stroke. *Curr. Neurovasc. Res.* 15 111–119. 10.2174/1567202615666180516120959 29766804

[B14] Choi H. I. Kim K. Lee J. Chang Y. Rhee H. Y. Park S. (2021). Relationship between Brain Tissue Changes and Blood Biomarkers of Cyclophilin A, Heme Oxygenase-1, and Inositol-Requiring Enzyme 1 in Patients with Alzheimer’s Disease. *Diagnostics* 11:740. 10.3390/diagnostics11050740 33919311 PMC8143350

[B15] Deane R. Du Yan S. Submamaryan R. K. LaRue B. Jovanovic S. Hogg E. (2003). RAGE mediates amyloid-beta peptide transport across the blood-brain barrier and accumulation in brain. *Nat. Med.* 9 907–913. 10.1038/nm890 12808450

[B16] Deane R. Sagare A. Zlokovic B. V. (2008). The role of the cell surface LRP and soluble LRP in blood-brain barrier Abeta clearance in Alzheimer’s disease. *Curr. Pharm. Des.* 14 1601–1605. 10.2174/138161208784705487 18673201 PMC2895311

[B17] Deane R. Wu Z. Sagare A. Davis J. Du Yan S. Hamm K. (2004). LRP/amyloid beta-peptide interaction mediates differential brain efflux of Abeta isoforms. *Neuron* 43 333–344. 10.1016/j.neuron.2004.07.017 15294142

[B18] Donahue J. E. Flaherty S. L. Johanson C. E. Duncan J. A. III Silverberg G. D. Miller M. C. (2006). RAGE, LRP-1, and amyloid-beta protein in Alzheimer’s disease. *Acta Neuropathol.* 112 405–415. 10.1007/s00401-006-0115-3 16865397

[B19] El-Ebidi A. M. Saleem T. H. Saadi M. G. E. Mahmoud H. A. Mohamed Z. Sherkawy H. S. (2020). Cyclophilin A (CyPA) as a Novel Biomarker for Early Detection of Diabetic Nephropathy in an Animal Model. *Diabetes Metab. Syndr. Obes.* 13 3807–3819. 10.2147/dmso.S260293 33116728 PMC7585800

[B20] Erkinjuntti T. (2003). Subcortical ischemic vascular disease and dementia. *Int. Psychogeriatr.* 15 23–26. 10.1017/s1041610203008925 16191213

[B21] Fazekas F. Chawluk J. B. Alavi A. Hurtig H. I. Zimmerman R. A. (1987). MR signal abnormalities at 1.5 T in Alzheimer’s dementia and normal aging. *AJR Am. J. Roentgenol.* 149 351–356. 10.2214/ajr.149.2.351 3496763

[B22] Halliday M. R. Rege S. V. Ma Q. Zhao Z. Miller C. A. Winkler E. A. (2016). Accelerated pericyte degeneration and blood-brain barrier breakdown in apolipoprotein E4 carriers with Alzheimer’s disease. *J. Cereb. Blood Flow Metab.* 36 216–227. 10.1038/jcbfm.2015.44 25757756 PMC4758554

[B23] Hilal S. Liu S. Wong T. Y. Vrooman H. Cheng C. Y. Venketasubramanian N. (2021). White matter network damage mediates association between cerebrovascular disease and cognition. *J. Cereb. Blood Flow Metab.* 41 1858–1872. 10.1177/0271678x21990980 33530830 PMC8327109

[B24] Horstmann S. Budig L. Gardner H. Koziol J. Deuschle M. Schilling C. (2010). Matrix metalloproteinases in peripheral blood and cerebrospinal fluid in patients with Alzheimer’s disease. *Int. Psychogeriatr.* 22 966–972. 10.1017/s1041610210000827 20561382

[B25] Jack C. R. Jr. Albert M. S. Knopman D. S. McKhann G. M. Sperling R. A. Carrillo M. C. (2011). Introduction to the recommendations from the National Institute on Aging-Alzheimer’s Association workgroups on diagnostic guidelines for Alzheimer’s disease. *Alzheimers Dement.* 7 257–262. 10.1016/j.jalz.2011.03.004 21514247 PMC3096735

[B26] Jia L. Quan M. Fu Y. Zhao T. Li Y. Wei C. (2020). Dementia in China: Epidemiology, clinical management, and research advances. *Lancet Neurol.* 19 81–92. 10.1016/s1474-4422(19)30290-x 31494009

[B27] Kao H. W. Lee K. W. Chen W. L. Kuo C. L. Huang C. S. Tseng W. M. (2015). Cyclophilin A in Ruptured Intracranial Aneurysm: A Prognostic Biomarker. *Medicine* 94:e1683. 10.1097/md.0000000000001683 26426668 PMC4616861

[B28] Lachén-Montes M. Íñigo-Marco I. Cartas-Cejudo P. Fernández-Irigoyen J. Santamaría E. (2021). Olfactory Bulb Proteomics Reveals Widespread Proteostatic Disturbances in Mixed Dementia and Guides for Potential Serum Biomarkers to Discriminate Alzheimer Disease and Mixed Dementia Phenotypes. *J. Pers. Med.* 11:503. 10.3390/jpm11060503 34204996 PMC8227984

[B29] Lam S. Lipton R. B. Harvey D. J. Zammit A. R. Ezzati A. (2021). White matter hyperintensities and cognition across different Alzheimer’s biomarker profiles. *J. Am. Geriatr. Soc.* 69 1906–1915. 10.1111/jgs.17173 33891712 PMC8456365

[B30] Li M. Sun H. Shen T. Xue S. Zhao Y. Leng B. (2021). Increased serum levels of cyclophilin a and matrix metalloproteinase-9 are associated with cognitive impairment in patients with obstructive sleep apnea. *Sleep Med.* 93 75–83. 10.1016/j.sleep.2021.10.009 34857483

[B31] Lorenzl S. Albers D. S. Relkin N. Ngyuen T. Hilgenberg S. L. Chirichigno J. (2003). Increased plasma levels of matrix metalloproteinase-9 in patients with Alzheimer’s disease. *Neurochem. Int.* 43 191–196. 10.1016/s0197-0186(03)00004-4 12689599

[B32] Lorenzl S. Buerger K. Hampel H. Beal M. F. (2008). Profiles of matrix metalloproteinases and their inhibitors in plasma of patients with dementia. *Int. Psychogeriatr.* 20 67–76. 10.1017/s1041610207005790 17697439

[B33] Ma Q. Zhao Z. Sagare A. P. Wu Y. Wang M. Owens N. C. (2018). Blood-brain barrier-associated pericytes internalize and clear aggregated amyloid-β42 by LRP1-dependent apolipoprotein E isoform-specific mechanism. *Mol. Neurodegener.* 13:57. 10.1186/s13024-018-0286-0 30340601 PMC6194676

[B34] Mawuenyega K. G. Sigurdson W. Ovod V. Munsell L. Kasten T. Morris J. C. (2010). Decreased clearance of CNS beta-amyloid in Alzheimer’s disease. *Science* 330:1774. 10.1126/science.1197623 21148344 PMC3073454

[B35] Montagne A. Barnes S. R. Sweeney M. D. Halliday M. R. Sagare A. P. Zhao Z. (2015). Blood-brain barrier breakdown in the aging human hippocampus. *Neuron* 85 296–302. 10.1016/j.neuron.2014.12.032 25611508 PMC4350773

[B36] Montagne A. Nation D. A. Sagare A. P. Barisano G. Sweeney M. D. Chakhoyan A. (2020). APOE4 leads to blood-brain barrier dysfunction predicting cognitive decline. *Nature* 581 71–76. 10.1038/s41586-020-2247-3 32376954 PMC7250000

[B37] Montagne A. Nikolakopoulou A. M. Huuskonen M. T. Sagare A. P. Lawson E. J. Lazic D. (2021). APOE4 accelerates advanced-stage vascular and neurodegenerative disorder in old Alzheimer’s mice via cyclophilin A independently of amyloid-β. *Nat. Aging* 1 506–520. 10.1038/s43587-021-00073-z 35291561 PMC8920485

[B38] Nation D. A. Sweeney M. D. Montagne A. Sagare A. P. D’Orazio L. M. Pachicano M. (2019). Blood-brain barrier breakdown is an early biomarker of human cognitive dysfunction. *Nat. Med.* 25 270–276. 10.1038/s41591-018-0297-y 30643288 PMC6367058

[B39] Nelson A. R. Sweeney M. D. Sagare A. P. Zlokovic B. V. (2016). Neurovascular dysfunction and neurodegeneration in dementia and Alzheimer’s disease. *Biochim. Biophys. Acta* 1862 887–900. 10.1016/j.bbadis.2015.12.016 26705676 PMC4821735

[B40] Neto E. Allen E. A. Aurlien H. Nordby H. Eichele T. (2015). EEG Spectral Features Discriminate between Alzheimer’s and Vascular Dementia. *Front. Neurol.* 6:25. 10.3389/fneur.2015.00025 25762978 PMC4327579

[B41] Nigro P. Satoh K. O’Dell M. R. Soe N. N. Cui Z. Mohan A. (2011). Cyclophilin A is an inflammatory mediator that promotes atherosclerosis in apolipoprotein E-deficient mice. *J. Exp. Med.* 208 53–66. 10.1084/jem.20101174 21173104 PMC3023134

[B42] Nikolakopoulou A. M. Wang Y. Ma Q. Sagare A. P. Montagne A. Huuskonen M. T. (2021). Endothelial LRP1 protects against neurodegeneration by blocking cyclophilin A. *J. Exp. Med.* 218:e20202207. 10.1084/jem.20202207 33533918 PMC7863706

[B43] O’Brien J. T. Thomas A. (2015). Vascular dementia. *Lancet* 386 1698–1706. 10.1016/s0140-6736(15)00463-8 26595643

[B44] Pan P. Zhao H. Zhang X. Li Q. Qu J. Zuo S. (2020). Cyclophilin a signaling induces pericyte-associated blood-brain barrier disruption after subarachnoid hemorrhage. *J. Neuroinflammation* 17:16. 10.1186/s12974-020-1699-6 31926558 PMC6954572

[B45] Park M. Moon W. J. (2016). Structural MR Imaging in the Diagnosis of Alzheimer’s Disease and Other Neurodegenerative Dementia: Current Imaging Approach and Future Perspectives. *Korean J. Radiol.* 17 827–845. 10.3348/kjr.2016.17.6.827 27833399 PMC5102911

[B46] Quinn K. A. Grimsley P. G. Dai Y. P. Tapner M. Chesterman C. N. Owensby D. A. (1997). Soluble low density lipoprotein receptor-related protein (LRP) circulates in human plasma. *J. Biol. Chem.* 272 23946–23951. 10.1074/jbc.272.38.23946 9295345

[B47] Ramachandran S. Venugopal A. Kutty V. R. Chitrasree V. Mullassari A. Pratapchandran N. S. (2014). Plasma level of cyclophilin A is increased in patients with type 2 diabetes mellitus and suggests presence of vascular disease. *Cardiovasc. Diabetol.* 13:38. 10.1186/1475-2840-13-38 24502618 PMC3922405

[B48] Rath D. von Ungern-Sternberg S. Heinzmann D. Sigle M. Monzien M. Horstmann K. (2020). Platelet surface expression of cyclophilin A is associated with increased mortality in patients with symptomatic coronary artery disease. *J. Thromb. Haemost.* 18 234–242. 10.1111/jth.14635 31519036

[B49] Redell J. B. Zhao J. Dash P. K. (2007). Acutely increased cyclophilin a expression after brain injury: A role in blood-brain barrier function and tissue preservation. *J. Neurosci. Res.* 85 1980–1988. 10.1002/jnr.21324 17461417

[B50] Rempe R. G. Hartz A. M. S. Bauer B. (2016). Matrix metalloproteinases in the brain and blood-brain barrier: Versatile breakers and makers. *J. Cereb. Blood Flow Metab.* 36 1481–1507. 10.1177/0271678x16655551 27323783 PMC5012524

[B51] Sagare A. Deane R. Bell R. D. Johnson B. Hamm K. Pendu R. (2007). Clearance of amyloid-beta by circulating lipoprotein receptors. *Nat. Med.* 13 1029–1031. 10.1038/nm1635 17694066 PMC2936449

[B52] Sagare A. P. Deane R. Zetterberg H. Wallin A. Blennow K. Zlokovic B. V. (2011). Impaired lipoprotein receptor-mediated peripheral binding of plasma amyloid-β is an early biomarker for mild cognitive impairment preceding Alzheimer’s disease. *J Alzheimers Dis.* 24 25–34. 10.3233/jad-2010-101248 21157031 PMC4096563

[B53] Sagare A. P. Deane R. Zlokovic B. V. (2012). Low-density lipoprotein receptor-related protein 1: A physiological Aβ homeostatic mechanism with multiple therapeutic opportunities. *Pharmacol. Ther.* 136 94–105. 10.1016/j.pharmthera.2012.07.008 22820095 PMC3432694

[B54] Satoh K. Fukumoto Y. Sugimura K. Miura Y. Aoki T. Nochioka K. (2013). Plasma cyclophilin A is a novel biomarker for coronary artery disease. *Circ. J.* 77 447–455. 10.1253/circj.cj-12-0805 23138189

[B55] Satoh K. Nigro P. Zeidan A. Soe N. N. Jaffré F. Oikawa M. (2011). Cyclophilin A promotes cardiac hypertrophy in apolipoprotein E-deficient mice. *Arterioscler. Thromb. Vasc. Biol.* 31 1116–1123. 10.1161/atvbaha.110.214601 21330604 PMC3085960

[B56] Seizer P. Fuchs C. Ungern-Sternberg S. N. Heinzmann D. Langer H. Gawaz M. (2016). Platelet-bound cyclophilin A in patients with stable coronary artery disease and acute myocardial infarction. *Platelets* 27 155–158. 10.3109/09537104.2015.1051466 26084004

[B57] Seizer P. Schönberger T. Schött M. Lang M. R. Langer H. F. Bigalke B. (2010). EMMPRIN and its ligand cyclophilin A regulate MT1-MMP, MMP-9 and M-CSF during foam cell formation. *Atherosclerosis* 209 51–57. 10.1016/j.atherosclerosis.2009.08.029 19758589

[B58] Seizer P. Ungern-Sternberg S. N. Schönberger T. Borst O. Münzer P. Schmidt E. M. (2015). Extracellular cyclophilin A activates platelets via EMMPRIN (CD147) and PI3K/Akt signaling, which promotes platelet adhesion and thrombus formation in vitro and in vivo. *Arterioscler. Thromb. Vasc. Biol.* 35 655–663. 10.1161/atvbaha.114.305112 25550208

[B59] Shackleton B. Ringland C. Abdullah L. Mullan M. Crawford F. Bachmeier C. (2019). Influence of Matrix Metallopeptidase 9 on Beta-Amyloid Elimination Across the Blood-Brain Barrier. *Mol. Neurobiol.* 56 8296–8305. 10.1007/s12035-019-01672-z 31209784 PMC6842100

[B60] Shibata M. Yamada S. Kumar S. R. Calero M. Bading J. Frangione B. (2000). Clearance of Alzheimer’s amyloid-ss(1-40) peptide from brain by LDL receptor-related protein-1 at the blood-brain barrier. *J. Clin. Invest.* 106 1489–1499. 10.1172/jci10498 11120756 PMC387254

[B61] Storck S. E. Meister S. Nahrath J. Meißner J. N. Schubert N. Di Spiezio A. (2016). Endothelial LRP1 transports amyloid-β(1-42) across the blood-brain barrier. *J. Clin. Invest.* 126 123–136. 10.1172/jci81108 26619118 PMC4701557

[B62] Tachibana H. Washida K. Kowa H. Kanda F. Toda T. (2016). Vascular Function in Alzheimer’s Disease and Vascular Dementia. *Am. J. Alzheimers Dis. Other Demen.* 31 437–442. 10.1177/1533317516653820 27284205 PMC10852864

[B63] Tachibana M. Holm M. L. Liu C. C. Shinohara M. Aikawa T. Oue H. (2019). APOE4-mediated amyloid-β pathology depends on its neuronal receptor LRP1. *J. Clin. Invest.* 129 1272–1277. 10.1172/jci124853 30741718 PMC6391135

[B64] Uemura M. T. Maki T. Ihara M. Lee V. M. Y. Trojanowski J. Q. (2020). Brain Microvascular Pericytes in Vascular Cognitive Impairment and Dementia. *Front. Aging Neurosci.* 12:80. 10.3389/fnagi.2020.00080 32317958 PMC7171590

[B65] Ueno M. Chiba Y. Murakami R. Matsumoto K. Fujihara R. Uemura N. (2019). Disturbance of Intracerebral Fluid Clearance and Blood-Brain Barrier in Vascular Cognitive Impairment. *Int. J. Mol. Sci.* 20:2600. 10.3390/ijms20102600 31137875 PMC6566824

[B66] Wang J. Gu B. J. Masters C. L. Wang Y. J. (2017). A systemic view of Alzheimer disease - insights from amyloid-β metabolism beyond the brain. *Nat. Rev. Neurol.* 13 612–623. 10.1038/nrneurol.2017.111 28960209

[B67] Wang J. J. Wu S. B. Cheng H. L. Yang C. Chen Y. Yang J. (2020). [Effect of moxibustion on expression of RAGE and LRP-1 and neuronal ultrastructure of frontal cortex and hippocampus in vascular dementia rats]. *Zhen Ci Yan Jiu* 45 33–39. 10.13702/j.1000-0607.1902956 32144906

[B68] Xiang Y. Bu X. L. Liu Y. H. Zhu C. Shen L. L. Jiao S. S. (2015). Physiological amyloid-beta clearance in the periphery and its therapeutic potential for Alzheimer’s disease. *Acta Neuropathol.* 130 487–499. 10.1007/s00401-015-1477-1 26363791 PMC4575389

[B69] Yamazaki Y. Kanekiyo T. (2017). Blood-Brain Barrier Dysfunction and the Pathogenesis of Alzheimer’s Disease. *Int. J. Mol. Sci.* 18:1965. 10.3390/ijms18091965 28902142 PMC5618614

[B70] Yang Y. Estrada E. Y. Thompson J. F. Liu W. Rosenberg G. A. (2007). Matrix metalloproteinase-mediated disruption of tight junction proteins in cerebral vessels is reversed by synthetic matrix metalloproteinase inhibitor in focal ischemia in rat. *J. Cereb. Blood Flow Metab.* 27 697–709. 10.1038/sj.jcbfm.9600375 16850029

[B71] Zlokovic B. V. (2011). Neurovascular pathways to neurodegeneration in Alzheimer’s disease and other disorders. *Nat. Rev. Neurosci.* 12 723–738. 10.1038/nrn3114 22048062 PMC4036520

