# Supplementary material for: Contribution of blood-brain barrier-related blood-borne factors for Alzheimer’s disease vs. vascular dementia diagnosis: A pilot study
Source: Front Neurosci. 2022 Aug 8;16:949129. doi: 10.3389/fnins.2022.949129 (PMC9393528; doi:10.3389/fnins.2022.949129)
Supplement: Supplementary file 1 [file Data_Sheet_1.docx]

Supplementary Material

# Supplementary methods

The inclusion and exclusion criteria for AD and VD：

## Alzheimer’s disease

#### *1.1.1 Inclusion criteria*

1. Age≥ 40 years, female or male;
2. With an education of primary school (included) or above;
3. Meet the diagnostic criteria for dementia in *The Diagnostic and Statistical Manual of Mental Disorders* (Edition 4);
4. Meet the *NIA-AA of Probable Alzheimer Dementia;*
5. MMES scores < 20 (primary school) or < 24 (junior school or above);
6. CT or MRI scans within 12 months prior to screening showed no evidence of infective infarction or other focal injury and no clinical symptoms but allowed lacunar infarction in a non-critical brain region and was not considered to influence cognitive impairment.

### *Exclusion Criteria*

1. Subject with family history of dementia;
2. Subject with history of stroke and focal signs of nervous system, and imaging findings consistent with small vascular disease (Fazekas score ≥ 2 points);
3. Other neurological diseases that may cause cognitive impairment (such as depression, brain tumor, Parkinson's disease, metabolic encephalopathy, encephalitis, multiple sclerosis, epilepsy, brain trauma, and normal intracranial pressure hydrocephalus.);
4. Other systemic diseases that may cause cognitive impairment (such as liver, renal and thyroid insufficiency, severe anemia, folic acid or vitamin B12 deficiency, syphilis, HIV infection, alcohol and drug abuse.);
5. Mental and neurodevelopmental retardation;
6. Contraindications to MRI;
7. Suffer from a disease that cannot complete cognitive examination;
8. Refuse to draw blood.

## 1.2 Subcortical ischemic vascular disease

### *1.2.1 Inclusion criteria*

1. Age≥ 40 years, female or male;
2. With an education of primary school (included) or above;
3. Meet the modification of *NINDS*-*AIREN Criteria of Probable Vascular Dementia*:
4. Cognitive symptoms (the following two points should be met)

Executive function abnormality: affecting the establishment of goals, planning, organization, program implementation, transformation, and maintenance of abstraction.

Memory impairment (can be mild): mainly recall function impairment, and recognition ability relatively intact, moderate, or mild forgetting, can be improved by cueing recall; (Compared with the past, functional decline, complex occupational and social activities are impaired, and the decline in function is not due to the influence of physical function caused by cerebrovascular disease).

1. Cerebrovascular disease (both of the following points should be met)

Brain imaging demonstrated the presence of associated cerebrovascular disease (white matter lesion: paraventricular > 10mm, deep white matter > 25mm, or extensive white matter change.

History or present presence of neurological signs consistent with subcortical cerebrovascular disease (e.g., mild hemiplegia, Babinski sign, facial palsy below palpebral cleft, hypoesthesia, dysarthria, abnormal gait, and extrapyramidal signs).

1. Clinical features supporting the diagnosis of SIVD

Symptoms of upper motor neuron involvement, such as asymmetrical reflex and ataxia in hemiplegia; Early gait abnormalities (small gait initiation difficulty ataxic apraxia Parkinson's gait); Instability and falls for no apparent reason; Urinary diseases unexplained frequency of urination and urgency with other urinary symptoms; Dysarthria dysphagia, extrapyramidal symptoms (decreased motor function and rigidity); Behavioral and psychiatric symptoms.

### *1.2.2 Exclusion Criteria*

1. Hippocampal or entorhinal cortex atrophy beyond aging;
2. Other neurological diseases that may cause cognitive impairment (such as depression, brain tumor, Parkinson's disease, metabolic encephalopathy, encephalitis, multiple sclerosis, epilepsy, brain trauma, and normal intracranial pressure hydrocephalus.);
3. Other systemic diseases that may cause cognitive impairment (such as liver, renal and thyroid insufficiency, severe anemia, folic acid or vitamin B12 deficiency, syphilis, HIV infection, alcohol and drug abuse.);
4. Mental and neurodevelopmental retardation;
5. Contraindications to MRI;
6. Suffer from a disease that cannot complete cognitive examination;
7. Refuse to draw blood.

# Supplementary Figures and Tables

## Supplementary Figures

Fazekas score：

Score 0


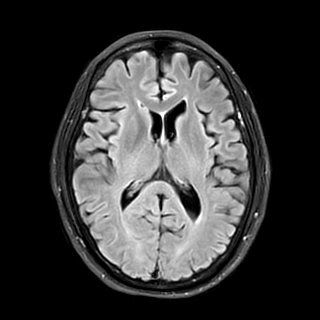

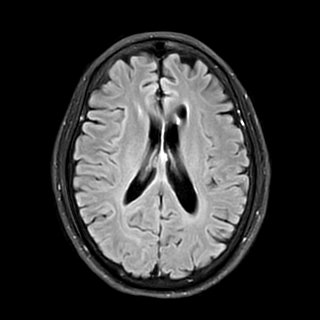


Score 1


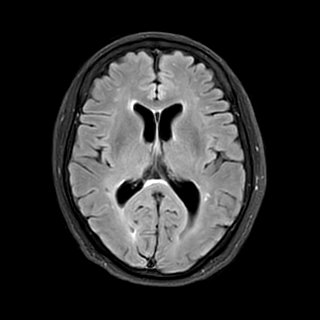

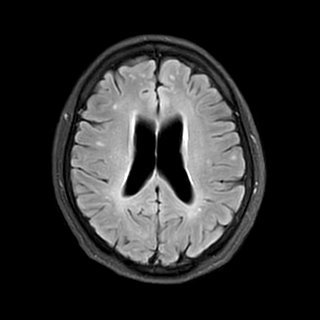


Score 2


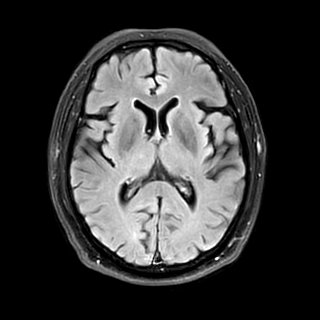

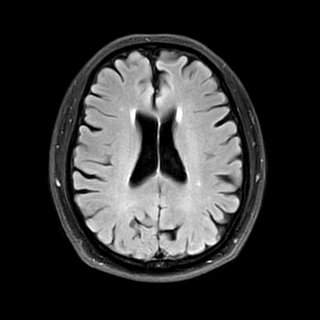


Score 3


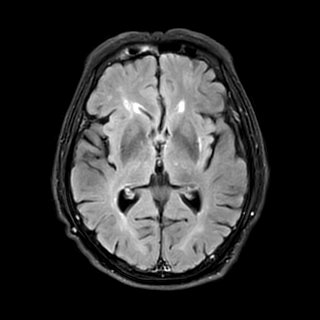

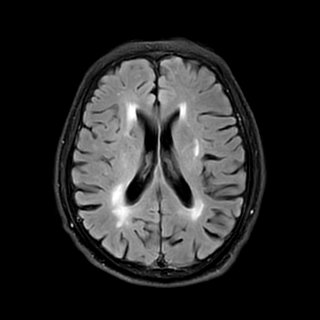


Score 4


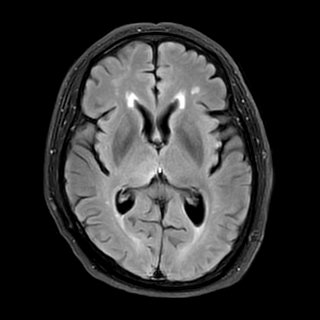

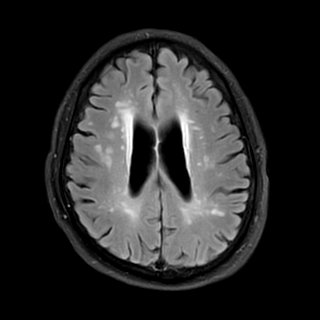


Score 5


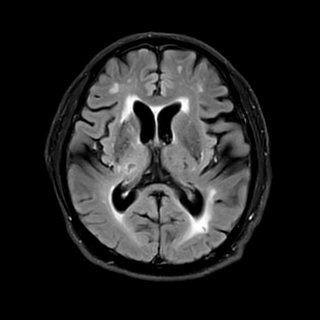

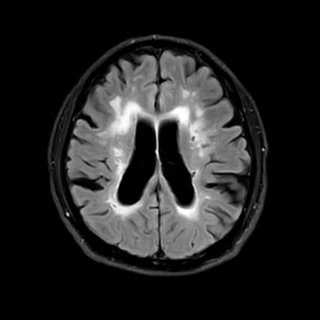


Score 6


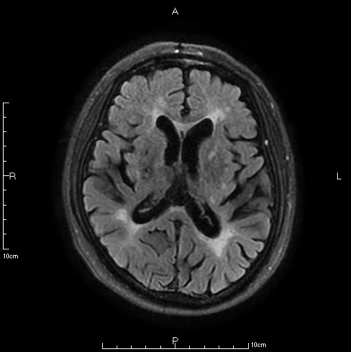

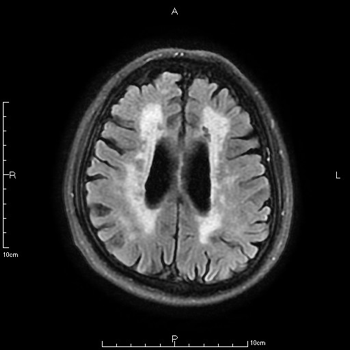


MTA score:

Score 0 Score 1 Score 2 Score 3 Score 4


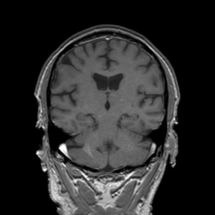

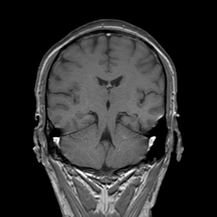

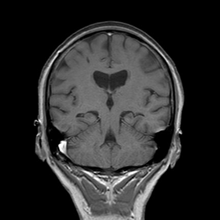

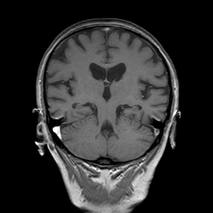

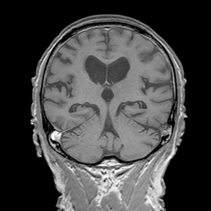


**Supplementary Figure 1.** The typical images of MTA/white matter hyperintensity from patients.


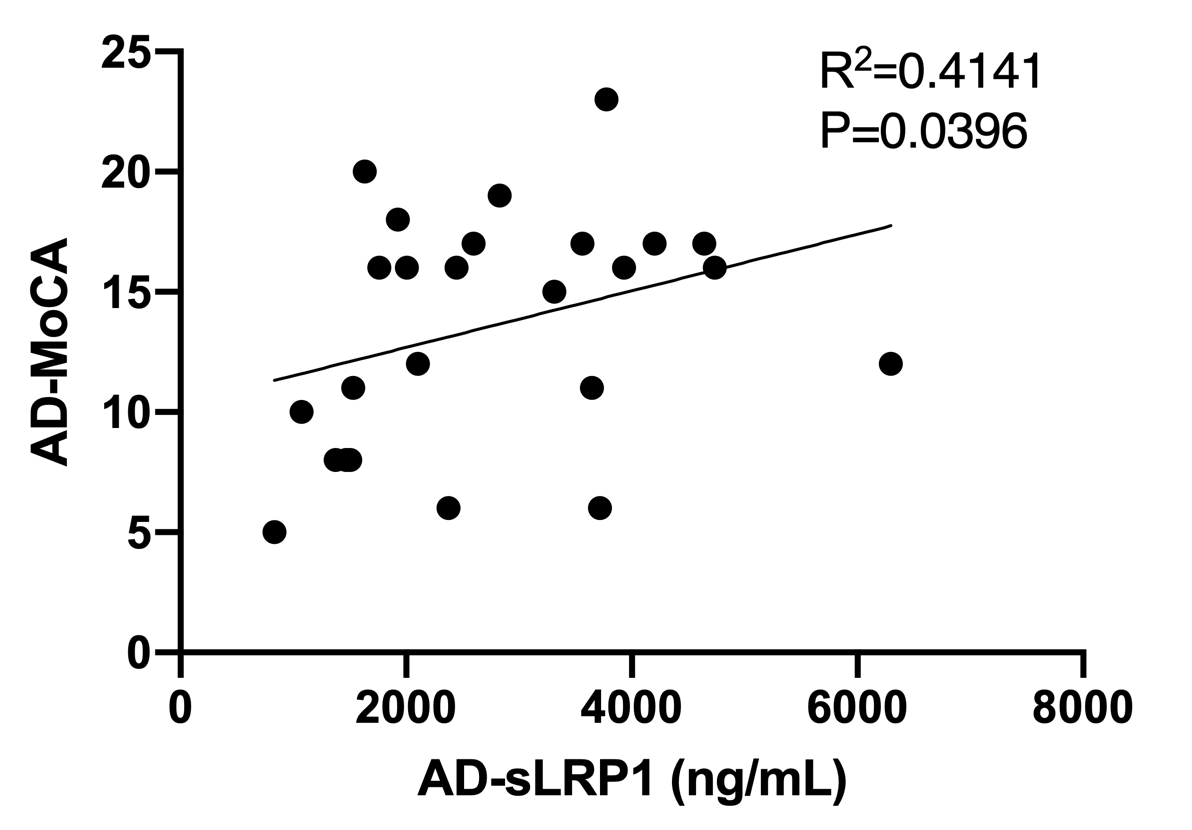

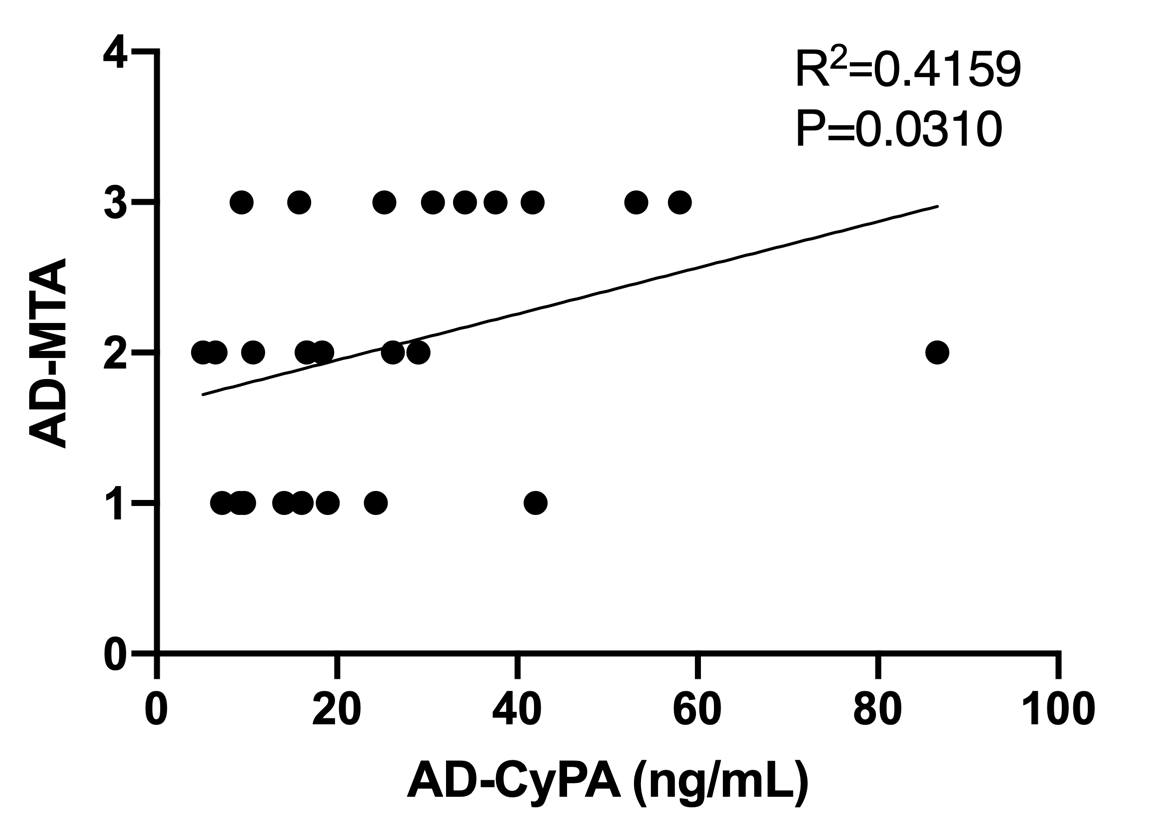

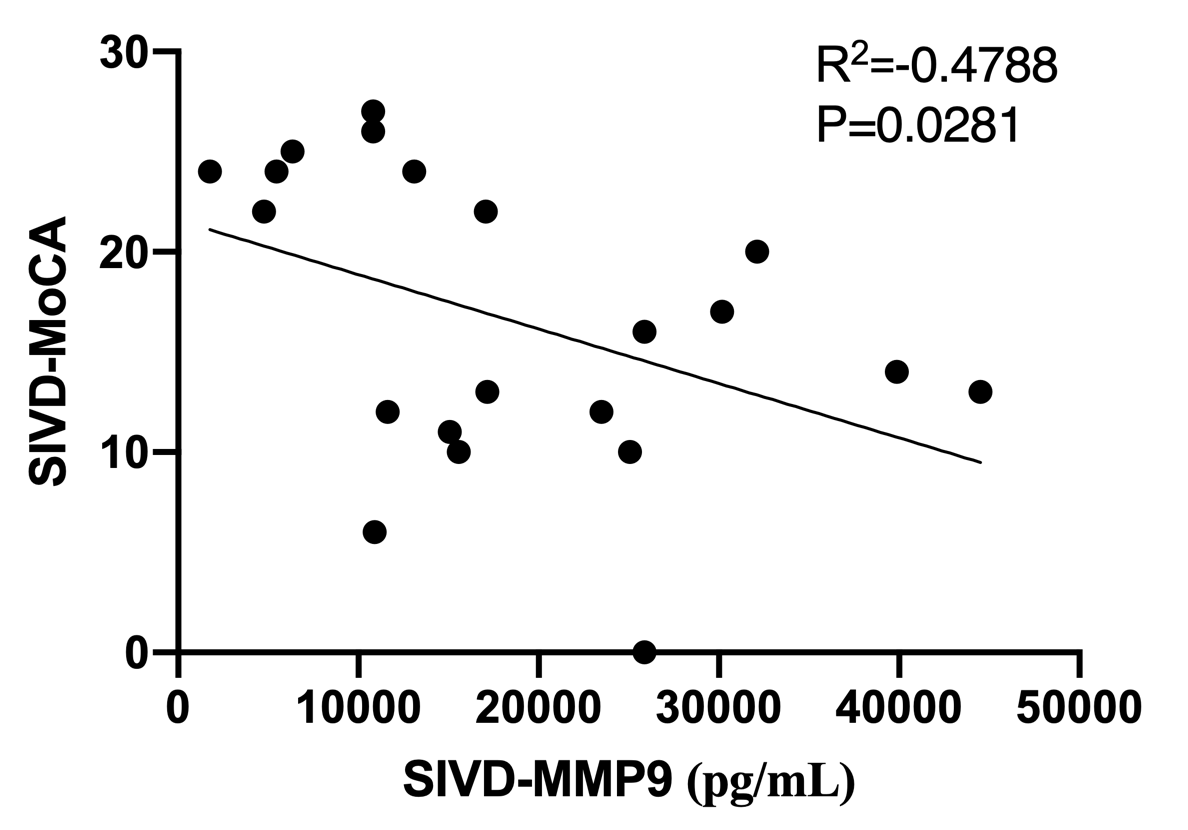


**Supplementary Figure 2.** Correlation of plasma biomarker levels with cognitive scores and imaging parameters by linear regression.

## Supplementary Tables

## Supplementary Table 1. Correlation of plasma biomarker levels with other blood test results or vascular risks

| **Factors** | **sLRP1** | | **CyPA** | | **MMP9** | |
| --- | --- | --- | --- | --- | --- | --- |
|  | **r** | ***P*** | **r** | ***P*** | **r** | ***P*** |
| **The whole sample** |  |  |  |  |  |  |
| LDL | -0.254 | 0.026^*^ | 0.052 | 0.655 | 0.000 | 0.998 |
| HDL | -0.461 | 0.0001^*^ | -0.132 | 0.252 | -0.072 | 0.538 |
| TC | -0.301 | 0.008^*^ | -0.004 | 0.974 | -0.026 | 0.824 |
| TG | -0.289 | 0.011^*^ | -0.049 | 0.672 | 0.108 | 0.354 |
| Hypertension | 0.134 | 0.235 | 0.032 | 0.778 | -0.005 | 0.964 |
| diabetes | 0.019 | 0.869 | 0.426 | 0.0001^*^ | 0.157 | 0.168 |
| hyperlipidemia | 0.024 | 0.829 | 0.269 | 0.016^*^ | 0.159 | 0.161 |
| **The AD groups** |  |  |  |  |  |  |
| LDL | -0.166 | 0.427 | 0.300 | 0.145 | 0.022 | 0.916 |
| HDL | -0.535 | 0.006^*^ | -0.296 | 0.151 | -0.094 | 0.655 |
| TC | -0.225 | 0.280 | 0.199 | 0.340 | -0.005 | 0.980 |
| TG | 0.419 | 0.037^*^ | 0.108 | 0.608 | 0.163 | 0.436 |
| Hypertension | 0.036 | 0.860 | 0.228 | 0.262 | -0.151 | 0.463 |
| diabetes | 0.110 | 0.594 | 0.292 | 0.147 | -0.049 | 0.813 |
| hyperlipidemia | -0.008 | 0.969 | 0.313 | 0.119 | 0.361 | 0.070 |
| **The SIVD groups** |  |  |  |  |  |  |
| LDL | -0.346 | 0.090 | 0.126 | 0.548 | 0.206 | 0.335 |
| HDL | -0.498 | 0.011^*^ | 0.061 | 0.771 | -0.242 | 0.255 |
| TC | -0.406 | 0.044^*^ | 0.059 | 0.779 | 0.137 | 0.525 |
| TG | 0.100 | 0.633 | -0.271 | 0.190 | 0.005 | 0.982 |
| Hypertension | 0.148 | 0.462 | -0.276 | 0.164 | -0.301 | 0.136 |
| diabetes | -0.119 | 0.553 | 0.412 | 0.033^*^ | 0.214 | 0.294 |
| hyperlipidemia | -0.110 | 0.584 | 0.245 | 0.218 | 0.163 | 0.427 |

**Note:** adjusted for age, sex, and education. ^∗^*p* < 0.05.

## Supplementary Table 2. Correlation of plasma biomarker levels with the symptoms of cognitive domains

| **Factors** | **sLRP1** | | **CyPA** | | **MMP9** | |
| --- | --- | --- | --- | --- | --- | --- |
|  | **r** | **P** | **r** | **P** | **r** | ***P*** |
| **The whole sample** |  |  |  |  |  |  |
| Memory | NA | NA | NA | NA | NA | NA |
| Language | 0.122 | 0.389 | 0.017 | 0.905 | 0.205 | 0.150 |
| Executive | 0.353 | 0.010^*^ | 0.327 | 0.018^*^ | 0.063 | 0.659 |
| Behaviour | -0.060 | 0.674 | 0.162 | 0.251 | 0.075 | 0.599 |
| Visuospatial | -0.288 | 0.038^*^ | -0.259 | 0.064 | -0.262 | 0.063 |
| **The AD groups** |  |  |  |  |  |  |
| Memory | NA | NA | NA | NA | NA | NA |
| Language | 0.093 | 0.650 | -0.040 | 0.846 | 0.333 | 0.096 |
| Executive | 0.295 | 0.144 | 0.191 | 0.350 | -0.110 | 0.593 |
| Behaviour | -0.260 | 0.900 | 0.260 | 0.200 | 0.410 | 0.037^*^ |
| Visuospatial | -0.251 | 0.216 | -0.374 | 0.059 | 0.005 | 0.980 |
| **The SIVD groups** |  |  |  |  |  |  |
| Memory | NA | NA | NA | NA | NA | NA |
| Language | 0.024 | 0.906 | -0.110 | 0.594 | 0.042 | 0.843 |
| Executive | NA | NA | NA | NA | NA | NA |
| Behaviour | -0.067 | 0.743 | 0.088 | 0.668 | -0.184 | 0.377 |
| Visuospatial | -0.189 | 0.356 | 0.111 | 0.591 | -0.513 | 0.009^*^ |

**Note:** adjusted for age, sex, and education. ^∗^*p* < 0.05.

## Supplementary Table 3. Logistic regression analysis of plasma biomarker levels

| **Factors** | **AD vs NC** | | **SIVD vs NC** | | **AD vs SIVD** | |
| --- | --- | --- | --- | --- | --- | --- |
|  | **OR（95%CI）** | ***P*** | **OR（95%CI）** | ***P*** | **OR（95%CI）** | ***P*** |
| sLRP1 | 1.000（0.999~1.000） | 0.501 | 1.000（1.000~1.000） | 0.086 | 0.999（0.999~1.000） | 0.059 |
| CyPA | 0.967（0.938~0.997） | 0.032* | 1.004（0.984~1.024） | 0.708 | 0.963（0.934~0.993） | 0.016* |
| MMP9 | 1.000（1.000~1.000） | 0.501 | 1.000（1.000~1.000） | 0.086 | 1.000（1.000~1.000） | 0.212 |

**Note:** adjusted for age, sex, and education. ^∗^*p* < 0.05.

## Supplementary Table 4. Logistic regression analysis of plasma biomarker levels

| **Factors** | **AD vs NC** | | **SIVD vs NC** | | **AD vs SIVD** | |
| --- | --- | --- | --- | --- | --- | --- |
|  | **OR（95%CI）** | ***P*** | **OR（95%CI）** | ***P*** | **OR（95%CI）** | ***P*** |
| sLRP1 | 1.000（1.000~1.001） | 0.486 | 1.000（1.000~1.001） | 0.064 | 1.001（1.000~1.001） | 0.051 |
| CyPA | 1.029（0.998~1.060） | 0.068 | 1.005（0.985~1.026） | 0.603 | 1.048（1.011~1.088） | 0.011* |
| MMP9 | 1.000（1.000~1.000） | 0.145 | 1.000（1.000~1.000） | 0.689 | 1.000（1.000~1.000） | 0.037* |

**Note:** adjusted for age, sex, education, hypertension, and TG. ^∗^*p* < 0.05.
